# Recent Advances in the Preparation of Block Copolymer Colloids and Porous Hydrogels Mediated by Emulsion Droplets

**DOI:** 10.3390/gels11110861

**Published:** 2025-10-28

**Authors:** Tengying Ma, Yining Liu, Yingying Wang, Nan Yan

**Affiliations:** 1 Research Institute for Scientific and Technological Innovation, College of Chemistry, Changchun Normal University, Changchun 130032, China; 2 School of Physics and Electronic Engineering, Shanxi Normal University, Taiyuan 030032, China

**Keywords:** anisotropic particles, emulsion gels, nanostructure control, self-assembly, stimulus-responsive materials, surfactant engineering

## Abstract

The versatility of emulsions as templates for fabricating functional materials has garnered significant attention in recent decades. Emulsions with tailored geometries provide a powerful platform for designing and synthesizing polymeric materials with diverse functionalities. This review summarizes recent advances in emulsion-mediated fabrication of block copolymer (BCP) functional colloids and emulsion-templated construction of gel emulsion and porous hydrogels. Key topics include the generation of high-quality, uniform emulsion droplets, control over the shape and internal nanostructure of BCP colloids, and strategies for constructing polymeric gels and other porous functional materials using gel emulsion as templates. Furthermore, the intrinsic properties of polymers can be pre-engineered with specific stimulus-responsive functionalities prior to the fabrication of polymeric microparticles or porous hydrogels, thus imparting novel and targeted functionalities to the resulting assemblies and porous networks. This study can help in developing crucial strategies and in identifying pathways for the rational design of novel multifunctional materials with applications in drug delivery, sensing, and catalysis.

## 1. Introduction

Recent advances in materials science and nanotechnology have established emulsion-mediated fabrication as a highly promising strategy for producing novel block copolymer (BCP) colloidal microparticles [1,2,3,4,5]. This approach utilizes flexible boundaries to create deformable emulsion droplets, which can be utilized as effective templates for engineering functional BCP materials with controllable internal nanostructures. The resulting materials show great potential for advanced applications in diverse fields, including photonic crystals [6,7], drug delivery and release [8,9], smart materials [10], catalysis [11,12,13,14], biosensing [15,16], magnetic materials [17], and energy materials [18,19,20,21,22,23]. In recent decades, emulsion droplet fabrication techniques have been continuously advancing, including methods such as ultrasonication, mechanical stirring, homogenization, microfluidics, template and Shirasu porous glass (SPG) membranes [24,25,26,27,28,29,30,31,32,33]. These methods can construct simple single-emulsion systems or complex double-emulsion systems. BCP assembled colloids were prepared within emulsion droplets. The emulsion interface directly contacts and interacts with the BCP blocks. Thus, the intrinsic properties of the BCP blocks, the interface characteristics, and their interactions are all critical to the shape and morphology of the final particles [34]. Therefore, modifying the properties of the blocks themselves (such as volume fraction and interactions between blocks) or adjusting the interface properties through surfactants or additives provides effective pathways for controlling the shape and internal phase-separated structures of self-assembled BCP colloids [35,36,37,38].

In addition, the amphiphilic BCPs containing both the hydrophobic and hydrophilic segments can also be utilized as superior emulsifiers and surfactants to prepare the gel emulsion [39,40]. Compared to conventional small molecules, BCPs have high molecular weight and controllable amphiphilicity. These properties prevent the fusion of gel emulsions. Consequently, BCPs provide more stable and excellent templates for fabricating hydrogels, microgels, and other porous functional materials. It is noteworthy that for amphiphilic BCPs utilized in stabilizing gel emulsion, the composition and volume ratio of the two blocks significantly influence the thermal stability and rheological properties of the gel emulsion [41]. For the preparation of hydrogels, the BCPs or the additional added polymeric monomer can be further crosslinked via chemical methods or photo-/temperature-induced reactions, thereby yielding polymeric hydrogels that combine high performance with excellent stability [42,43]. Alternatively, emulsion polymerization can be conducted within the emulsion, where the hydrophobic blocks aggregate via hydrophobic interactions, leading to the formation of hydrogels with enhanced storage modulus [44]. Furthermore, by precisely designing the properties and functionalities of the utilized BCPs at the segment level, a series of multifunctional hydrogels responsive to external stimuli can be obtained, offering a significant strategy and pathway for fabricating advanced multifunctional hydrogels [45,46].

While considerable efforts have been devoted to developing BCP-based microspheres and BCP-stabilized hydrogels, a systematic overview of the strategies for controlling microparticle architectures and fabricating hydrogels, along with the latest advances in their functional applications, remains imperative. This review first presents preparation methods of highly uniform emulsion droplets for fabricating BCP colloids with controllable shapes, with a focus on microfluidic technology and SPG membrane emulsification techniques. The influence of factors, including the inherent properties of polymer chains, the characteristics of surfactants and responsive surfactants, and responsive functional additives, has been systematically investigated for the preparation of the assembled BCP colloids. Subsequently, we present a series of methodologies for fabricating polymeric hydrogels and functional porous materials via gel emulsion templates, encompassing techniques such as emulsion polymerization, the use of BCP stabilizers, and subsequent crosslinking strategies. Finally, for the obtained functional materials, including polymer assemblies, polymer-based porous hydrogels and materials prepared by gel emulsion, their applications as well as the challenges and future prospects in this field are discussed.

## 2. Preparation of Emulsion Droplets

Currently, there are numerous methods and techniques for preparing 3D confined emulsion droplets, including ultrasonication, mechanical stirring, homogenization, SPG membrane emulsification, and microfluidics. Among all the emulsification strategies, SPG membrane emulsification and microfluidics offer outstanding advantages due to their ability to produce highly uniform and tunable droplet sizes, providing effective routes for creating monodisperse and adjustable emulsion systems.

### 2.1. Microfluidics Technique

Microfluidic technology has made significant advancements in constructing 3D emulsion droplets, primarily reflected in its precise control over droplet size, shape, structure, and functionality. Microfluidics can accurately control the flow of multiphase fluids. By utilizing different microchannel geometries (e.g., T-junctions, co-flow, and flow-focusing) and active control methods (such as electric fields), the composition and flow rate of fluids can be adjusted to achieve highly monodisperse and structurally complex droplets, including core–shell, multi-core, Janus, and controlled chamber structures [47,48]. The emulsions used for droplet generation in microfluidic devices can be categorized into four system structures: oil-in-water (O/W), water-in-oil (W/O), water-in-oil-in-water (W/O/W), and oil-in-water-in-oil (O/W/O) emulsions [49,50,51,52,53,54,55].

#### 2.1.1. Single Emulsions

**Figure 1:**
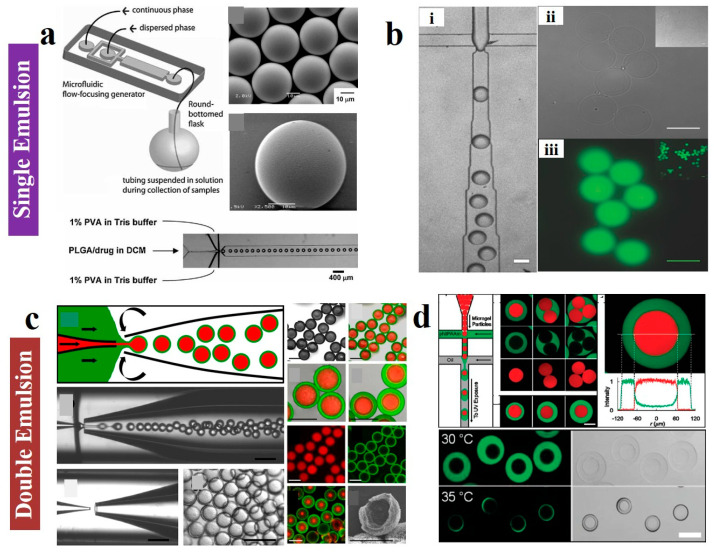
(**a**) Fabrication of microparticles using an O/W single-emulsion microfluidic approach. Reproduced with permission [49]. Copyright 2009, Wiley. (**b**) The alginate microcarriers prepared via W/O single-emulsion microfluidic approach. Scale bars are 50 μm. Reproduced with permission [51]. Copyright 2015, Wiley. (**c**) Microfluidic generation of W/O/W double emulsions using a co-flow capillary device and resultant core–shell particles featuring a solidified lipid shell and an aqueous core. Reproduced with permission [53]. Copyright 2013, ACS. (**d**) Schematic of the microfluidic device forming O/W/O microfluidic double emulsions. Reproduced with permission [55]. Copyright 2010, ACS.

Single emulsification refers to the process of dispersing one fluid as droplets within another immiscible fluid. Common forms of microfluidic single emulsions include O/W and W/O types. All three classic microfluidic geometries-co-flow, flow-focusing, and T-junction-can be utilized to generate single emulsions [56,57,58,59,60]. Among these, the co-flow geometry involves the dispersed phase flowing through an inner capillary into a coaxial stream of continuous phase moving in the same direction, exiting through a defined orifice. For example, Anderson et al. [49] used microfluidic devices (Figure 1a) to prepare biodegradable drug-loaded microparticles through generation of O/W single emulsion droplets containing poly(lactic-*co*-glycolic acid) and drug. Weitz et al. [51] showed the preparation of monodisperse alginate microgels from the W/O emulsion systems constructed by microfluidic technology (Figure 1b). This configuration is also widely employed in the fabrication of BCP particles [61]. Through extensive efforts aimed at synthesizing polymeric colloidal particles with precisely controlled shapes, sizes, and internal structures, 3D spherical confinement has emerged as a distinctive platform for such studies. Under 3D spherical confinement, BCP chains undergo heightened frustration due to the environmental constraints that prevent structural reorientation. The deformable, mobile interface of emulsion droplets enables spontaneous shape changes during self-assembly. Consequently, novel morphologies arise under 3D confinement, including particles featuring concentric lamellar (onion-like) and cylindrically coiled internal structures.

Based on 3D soft confinement, BCP particles with different shapes have been obtained, such as multilayer stacked pupa, hamburger, disks, convex lenses (CL), multicompartment, nanorings, Janus spheres, and nanocups [62,63,64,65,66,67,68,69]. For example, Guo et al. [70] successfully prepared poly(4-vinylpyridine)-*block*-polystyrene-*block*-poly(4-vinylpyridine) (P4VP-*b*-PS-*b*-P4VP) triblock copolymer particles using 3D soft confinement self-assembly with cetyltrimethylammonium bromide (CTAB) as the sole surfactant. They obtained onion-like particles with concentrically stacked PS and P4VP layers and an outermost P4VP layer, spherical particles with internally curved P4VP cylinders, CL-shaped particles with hexagonal P4VP cylinders and neutral interfaces, tulip-shaped particles with axially stacked PS and P4VP layers and neutral interfaces, and inverted onion-like particles with an outermost PS layer.

#### 2.1.2. Double Emulsions

Double emulsions are typically generated by integrating two distinct geometric structures. A commonly used microfluidic device for double emulsions comprises two circular capillaries aligned with their orifices facing each other, both encased within a square capillary. This configuration effectively combines co-flow and flow-focusing geometries. The resulting double emulsions can form W/O/W or O/W/O structures [56]. Among these, W/O/W emulsions have been extensively utilized in the fabrication of hollow polymer microcapsules [71]. By simultaneously introducing two internal fluids, multicomponent emulsions can be prepared [72]. Another device for preparing double emulsions combines co-flow and co-flow geometries, resulting in thinner-walled emulsion droplets [71]. Double emulsions can also be generated through a two-stage process using a T-junction structure. In the initial stage, the inner fluid was enveloped by the middle fluid, forming single-emulsion droplets. These droplets then advanced to a second generator where they were encapsulated by the second outer fluid, ultimately yielding double-emulsion droplets. For instance, based on the double emulsion preparation technique, Yang et al. [73] introduced cationic lipids during the primary emulsification step, which could self-assemble at the water-oil interface and electrostatically complexed with the nucleic acids, successfully trapping them within the inner aqueous core during the second emulsion and achieving high encapsulation efficiency (>95%). In addition, Weitz et al. [53] used microcapillary device to construct W/O/W double emulsions to form core–shell structure. Particularly, this device allowed controlled injection of three different fluids, with the internal (red) and intermediate (green) fluids being focused by the external fluid (white) hydrodynamics to form core–shell structured particles (Figure 1c). Furthermore, they [55] used a microfluidic device (Figure 1d) to form the O/W/O double emulsions, thus generating microgel capsules composed of two miscible but different layers. Therefore, microfluidic technology can be employed to fabricate high-quality monodisperse single and double emulsion systems.

### 2.2. The SPG Membrane Emulsification Technique

**Figure 2:**
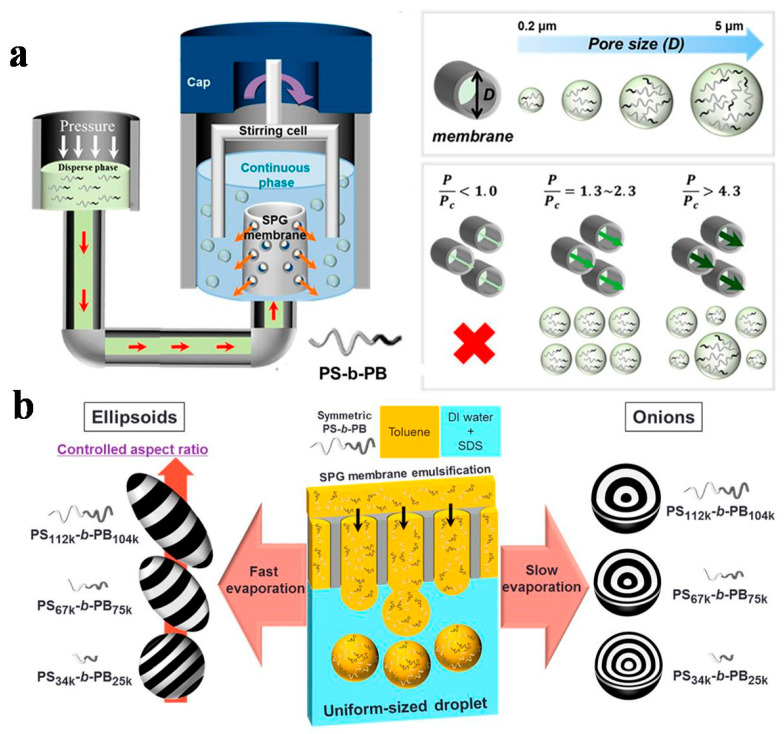
(**a**) SPG membrane emulsification process for controlling particle size and distribution through modulation of membrane pore size and operating pressure. Reproduced with permission [32]. Copyright 2015, ACS. (**b**) SPG membrane emulsification system for producing monodisperse PS-*b*-PB particles with morphology controlled by evaporation rate. Reproduced with permission [76]. Copyright 2017, ACS.

SPG is a microporous glass membrane made from CaO-Al_2_O_3_-B_2_O_3_-SiO_2_, where the CaO-B_2_O_3_ is removed through acid washing to form a uniform microporous structure. SPG membrane emulsification utilizes the surface chemical properties of the microporous membrane. By applying pressure, the dispersed phase (oil phase) is forced through a uniform microporous SPG glass membrane in either a “drop-by-drop” or “pre-mix” manner, entering the continuous aqueous phase (or conversely, the continuous oil phase) to generate monodisperse emulsion particles [63,74,75,76]. For instance, Yi et al. [32] successfully produced monodisperse BCP particles with sizes tunable from 200 nm to 5 μm via cross-flow membrane emulsification using an SPG membrane (Figure 2a). A toluene solution containing 0.01 g/mL PS-*block*-1,4-polybutadiene (PS-*b*-PB) was squeezed into an aqueous solution containing sodium dodecyl sulfate (SDS) surfactant through an SPG membrane at a given pressure. The agitator continuously applied shear force in the continuous phase, so that emulsion droplets could be separated from the pores. SDS quickly adsorbed on the generated toluene/water drop interface to stabilize the emulsion droplets. Subsequently, toluene slowly evaporated from the droplets, producing PS-*b*-PB BCP colloids. By optimizing operational parameters such as pressure, membrane pore size, and surfactant concentration, they achieved precise control over particle uniformity. Furthermore, the introduction of homopolymers led to the formation of coiled cylindrical morphologies inside the particles, with the resulting BCP assemblies exhibiting strong size dependence. This highlighted the effectiveness of their membrane-based approach in obtaining monodisperse systems. Shin et al. [76] synthesized PS-*b*-PB microparticles with distinctly different shapes and nanostructures through SPG membrane device, which was demonstrated to be primarily governed by the volatilization rate of organic reagents within the emulsion. The onion-like structure could be achieved under slower volatilization rates. This was because the slower rate provided sufficient time for polymer chains to rearrange, driven by the stronger affinity of PB for the emulsion interface, into this thermodynamically stable configuration. However, as the evaporation rate increased, the ellipsoidal colloids were kinetically obtained through the propagation of the BCP ordering (Figure 2b). Dai et al. [77] employed the tunable pore size of the membrane to fabricate a series of elongated PS-*block*-PB-*block*-poly(methyl methacrylate) (PS-*b*-PB-*b*-PMMA or SBM) elliptical microparticles with different sizes by combining SPG membrane emulsification with evaporation-induced confined assembly. By varying the pore size, they produced Janus disks with a controlled size distribution through the selective crosslinking and disassembly of SBM elliptical microparticles. They further transferred the Janus disks into water through the mild sulfonation of PS to polystyrene sulfonic acid, followed by verification of the Janus characteristics via cationic nanoparticle labeling. The experiments demonstrated that larger Janus disks were more effective in generating larger volumes of stable emulsion droplets. Thus, the SPG membrane emulsification technology has been widely used to prepare the single emulsion systems with tunable size distribution of the emulsion droplets.

To date, both microfluidic technology and SPG membrane emulsification have been capable of producing emulsion droplets with extremely uniform particle sizes, demonstrating significant potential for the further synthesis of functional microparticles. The SPG technique utilizes Shirasu porous glass membranes with uniform pore sizes, where the dispersed phase is extruded into the continuous phase under mild transmembrane pressure. Its advantages include simple equipment setup, ease of scale-up, and high monodispersity, though the emulsion droplet size is primarily constrained by the available membrane pore specifications and the applied pressure. In contrast, microfluidics employs precisely designed microchannel structures to actively control droplet breakup at the microscale, offering exceptional flexibility in droplet size tuning, strong capability for producing multiple emulsions, and excellent monodispersity. However, its throughput is generally limited. In summary, SPG membranes are more suitable for large-scale production of single emulsions with high uniformity requirements, while microfluidics demonstrates unique advantages in constructing complex emulsion structures and achieving precise size control.

## 3. Preparation of BCP Colloids and Polymeric Hydrogels Mediated by Emulsion Droplets

Based on the preparation of high-quality monodisperse emulsions, BCPs can be distributed at the interface or within the emulsion droplets by precisely regulating their intrinsic properties such as hydrophilicity/hydrophobicity and volume fraction. When BCPs are dispersed inside the emulsion droplets, stabilizers such as small molecules, polymers, or stimulus-responsive molecules are generally required to stabilize the emulsion droplets, thereby forming a stable emulsion system. BCP colloids can be obtained by controlling various factors during the solvent evaporation process. In contrast, when BCPs are distributed at the emulsion interface, they function as macromolecular surfactants, enabling high-quality stabilization of large amounts of emulsions. This facilitates the formation of concentrated gel emulsions with tunable rheological properties. Subsequent processes such as emulsion polymerization or selective crosslinking can yield porous functional materials like hydrogels. Therefore, emulsion-based preparation serves as a powerful templating strategy for the rational design of functional materials, including BCP colloids and porous hydrogels.

### 3.1. BCP Intrinsic Properties

**Figure 3:**
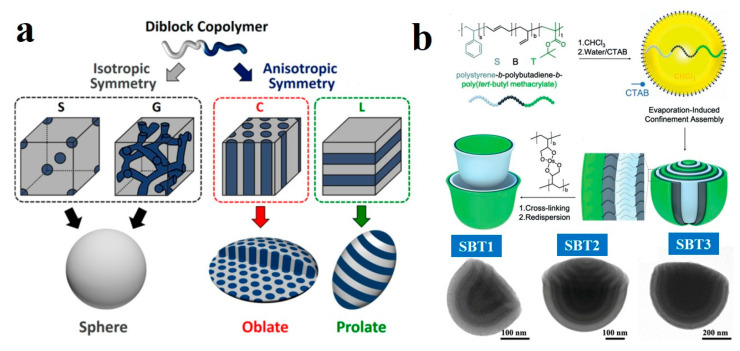
(**a**) Structures of self-assembled dBCPs: isotropic spherical and anisotropic oblate particles from confined emulsion droplets, and corresponding bulk phase separation structures. Reproduced with permission [84]. Copyright 2019, ACS. (**b**) TEM images of JNCs at 0° tilt angles of SBT1, SBT2 and SBT3. Reproduced with permission [85]. Copyright 2019, Wiley.

The phase segregation behavior of BCPs is a key factor governing both the overall shape of particles and their internal morphology. The resulting morphology is similar to their bulk counterparts. It is governed first by the volume fractions of the A and B blocks (*f*_A_ and *f*_B_), and second by the enthalpic repulsion between them. We quantify this repulsion using the parameter *χ*_AB_*N*. Among them, *χ*_AB_ represents the Flory-Huggins interaction parameter, while *N* denotes the degree of polymerization [35,78,79,80,81,82,83]. As shown in Figure 3a, Kim et al. [84] demonstrated that spherical particles were typically formed when diblock copolymers (dBCPs) with three-dimensionally isotropic symmetric structures (e.g., body-centered cubic and gyroid phases) were confined within emulsion droplets. Conversely, under neutral interfacial conditions (where polymer blocks had non-preferential interactions with the surface), microparticles with internally anisotropic microphases (e.g., cylindrical or lamellar) formed oblate or prolate shapes.

Compared to simple dBCPs, the influence of volume fraction on the structural transitions of triblock copolymers is more complex. Gröschel et al. [85] described the formation of shape- and surface-anisotropic Janus nanocups (JNCs) through evaporation-induced confined assembly. Under spherical confinement, microphase separation of the PS-*block*-PB-*block*-poly(*tert*-butyl methacrylate) (PS-*b*-PB-*b*-PtBMA or SBT) terpolymer occurred, which featured PS and PtBMA endblocks of comparable size and a variable PB midblock. This resulted in hemispherical microparticles exhibiting an internal concentric lamella-lamella (ll) morphology. Following cross-linking of the central PB block, these tulip-bulb-like particles disassembled into individual nanocups. The mechanical stability of the nanocup wall was found to correlate directly with the weight fraction of PB (*f*_PB_). As shown in transmission electron microscopy (TEM) images in Figure 3b, an increase in *f*_B_ was reflected by the thickening of the dark PB layers. At a low PB content (*f*_PB_ = 8 wt%, SBT1), the resulting JNCs possessed extremely thin walls that were prone to collapse. With *f*_PB_ increased to 22 wt% (SBT2), the nanocups became more flexible and typically remained closed; although those with smaller diameters could occasionally stay open. When *f*_PB_ was raised further to 43 wt% (SBT3), the cup walls became sufficiently rigid to maintain an open structure across a wide range of nanocup diameters.

**Figure 4:**
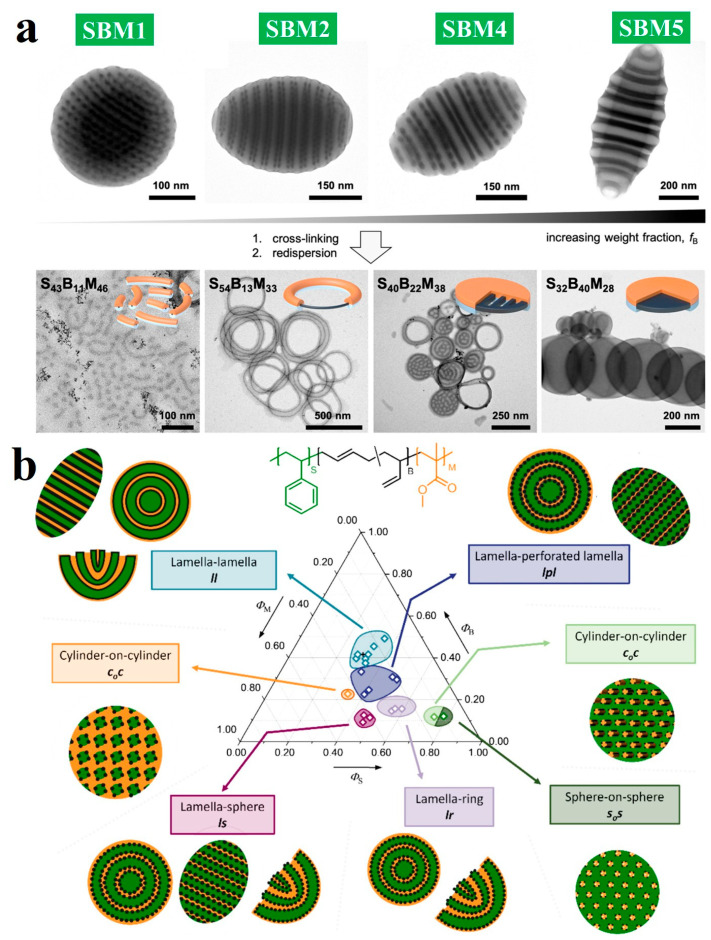
(**a**) The influence of *f*_B_ on the microstructures and Janus nanoparticles: SBM1 (*f*_B_ = 11 wt%), SBM2 (*f*_B_ = 13 wt%), SBM4 (*f*_B_ = 22 wt%), and SBM5 (*f*_B_ = 40 wt%). Reproduced with permission [86]. Copyright 2019, ACS. (**b**) Ternary microphase diagram of SBM in spherical confinement. Regions with different morphologies are indicated by different colors. Reproduced with permission [87]. Copyright 2025, Wiley.

Furthermore, studies were conducted on SBM triblock terpolymers featuring symmetric PS and PMMA blocks and a variable PB midblock [86]. At a PB weight fraction of *f*_PB_ = 11 wt%, a mixture of Janus nanospheres and short Janus nanorods was observed (Figure 4a). With SBM 2 (*f*_PB_ = 13 wt%), flexible Janus nanorings (JNRs) formed. Increasing *f*_PB_ to 22 wt% (SBM 4) resulted in JNRs with thicker, more defined cores and reduced flexibility, along with the appearance of perforated Janus disks. At *f*_PB_ = 40 wt% (SBM 5), the PB microphase evolved into continuous lamellae, leading to a PS/PB/PMMA lamellar-lamellar morphology and fully filled Janus disks. Gröschel et al. [87] systematically constructed the first ternary microphase diagram for the SBM triblock terpolymer system, which was achieved by conducting confined self-assembly of SMB polymer within 600 nm spherical droplets. Furthermore, dissipative particle dynamics simulations were combined with the experimental results. The work identified that twenty-two SBM triblock terpolymers with different volume fractions yielded six distinct particle morphologies, including lamellar-lamellar, lamella-perforated lamella, lamella-sphere, lamella-ring, sphere-on-sphere, and cylinder-on-cylinder structures (Figure 4b). The study established critical volume fraction thresholds for the PB block that trigger transitions among lamellar, perforated lamellar, cylindrical, and spherical internal structures. This work provided crucial theoretical and experimental guidance for the precise design and controlled fabrication of polymer microparticles with different volume fractions of triblock terpolymers.

In addition to the intrinsic volume ratio of the applied BCPs, the volume fraction can also be facilely adjusted through the incorporation of homopolymers that exhibit affinity for one of the blocks in the BCP. The interplay between different components and their spatial organization provides novel structures for multicompartment composite colloids with anisotropic properties. Ku et al. [88] conducted a detailed study on PS-*b*-P2VP BCPs, which achieved different particle morphologies by systematically adjusting the volume fraction, molecular weight, and ratio of each homopolymer. The total homopolymer volume fractions (*f*_hP_) were varied from 0 to 1, where *f*_hPS_ and *f*_hP2VP_ represented the respective volume fractions of PS and P2VP homopolymers within the polymer phase (i.e., *f*_hP_ = *f*_hPS_ + *f*_hP2VP_). At lower *f*_hP_ values (<0.6), the addition of homopolymers only induced subtle changes in the overall structure, resulting in elongated bullet-shaped particles with a slight increase in axial length. As *f*_hP_ approached 0.7, the colloids transitioned to dome-like shapes with more spherical contours. When *f*_hP_ reached 1.0 with only homopolymers, the colloids adopted a distinct Janus morphology.

### 3.2. Influence of Surfactants

In emulsion systems, surfactants play a crucial role in effectively stabilizing droplets. Surfactants not only enhance the stability of droplets within the emulsion but also offer a flexible approach to controlling interfacial interactions. Typically positioned at the oil-water interface, surfactants orient themselves with their hydrophilic ends directed toward the aqueous phase and their hydrophobic ends toward the oil phase [89,90,91,92,93]. This orientation promotes the gradual self-assembly of BCPs from the outer region to the inner region, significantly increasing the diversity of BCP self-assembled morphologies.

#### 3.2.1. Small-Molecule Surfactants

Particularly in the case of emulsion-solvent evaporation-induced confined assembly, surfactants play a dual role in stabilizing emulsion droplets and guiding the orientation of BCP chains at the interface. Yan et al. [90] introduced CTAB surfactant into water, where CTAB molecules adsorbed at the water/oil interface, significantly reducing the interfacial tension. The authors further measured the interfacial tension between PS/CTAB and P4VP/CTAB. When the CTAB concentration reached 12 mg/mL, the interfacial tensions dropped sharply to comparable values of 2.9 ± 0.2 and 2.8 ± 0.2 mN/m, respectively. This reduction prompted the deformation of the emulsion droplets, resulting in the formation of sheet-like particles. They [26] also utilized the mutual attraction between the ligands on gold nanoparticles and surfactants (CTAB and poly(vinyl alcohol) (PVA)) at the oil/water interface to precisely control the spatial positioning and arrangement of functional gold nanoparticles on BCP scaffolds, which hold significant importance for enhancing the performance of hybrid nanomaterials.

**Figure 5:**
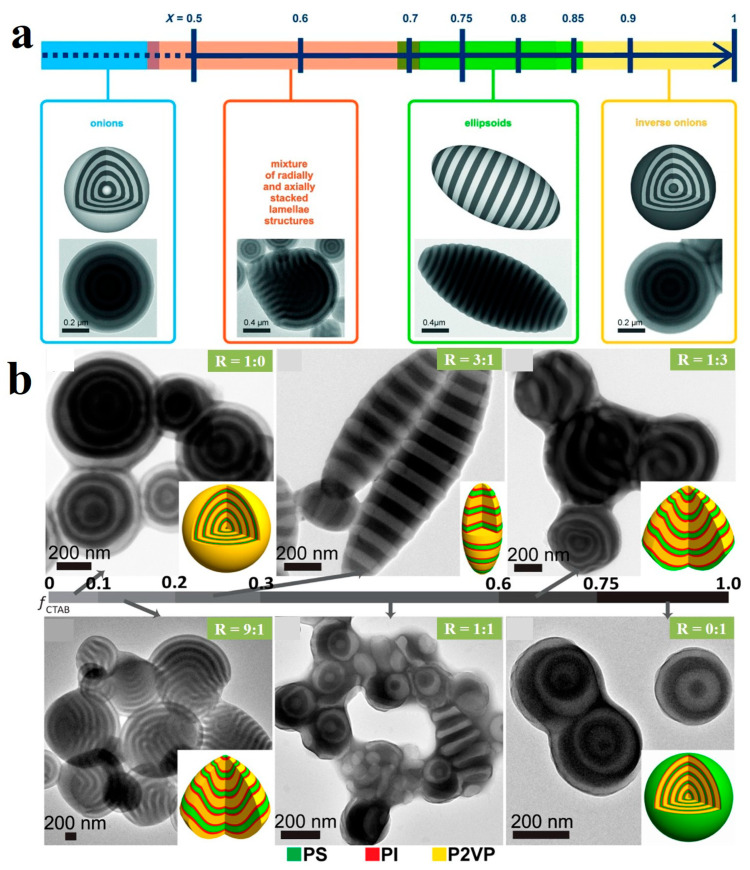
(**a**) Schematic of particle shape and internal morphology modulation in PS-*b*-P2VP BCPs via *x*_HO-CTAB_ variation in CTAB/HO-CTAB mixtures. Reproduced with permission [91]. Copyright 2014, Wiley. (**b**) TEM images of PS_40K_-*b*-PI_33K_-*b*-P4VP_87K_ particles obtained at different R. Reproduced with permission [93]. Copyright 2019, ACS.

To achieve precise control over the affinity and interactions at the emulsion interface with the respective blocks, the type and properties of surfactants can be tailored. This regulation makes it possible to effectively alter the interfacial interactions and the deposition sequence of the blocks during solvent evaporation. For example, Hawker et al. [91] designed a mixture of CTAB and hydroxylated CTAB (CTAB-OH) as surfactants to control the interaction between PS-*b*-P2VP BCPs and the surrounding medium, thereby constructing a neutral interface. By precisely regulating the mass fraction (*x*) of the two surfactants, the self-assembly process and the transformation of particle shapes were controlled. CTAB and CTAB-OH had strong affinities for PS and P2VP, respectively. Consequently, by adjusting the mass fraction ratios, a series of morphological transitions were observed. These included onion-like structures, spherical and nonspherical particles with mixed radially and axially stacked lamellae, as well as ellipsoids and reverse onion-like configurations (Figure 5a). Zhu et al. [93] achieved precise control over the morphology of PS-*block*-polyisoprene-*block*-P2VP (PS-*b*-PI-*b*-P2VP) ABC triblock BCPs by utilizing CTAB and PVA as surfactants. Through adjusting the different weight ratio of PVA to CTAB (R), morphological transitions were achieved among onion-like, bud-like, and pupa-like particles (Figure 5b). When R = 1:0, onion-like particles were obtained. At R = 9:1, bud-like particles formed, featuring terrace-like tips and onion-like hemispheres. A ratio of R = 3:1 yielded elliptical pupa-like particles composed of alternating stacked layers of PS, PI, and P2VP. Further increasing the mass ratio of CTAB, at R = 1:3, inverse bud-like particles were obtained, which consisted of terrace-like tips with alternating layers and inverse onion-like hemispheres with PS on the outermost layer. This structure was analogous to that of R = 9:1 but had an inverted internal structure. When only the CTAB surfactant was used, an inverse onion structure was obtained.

**Figure 6:**
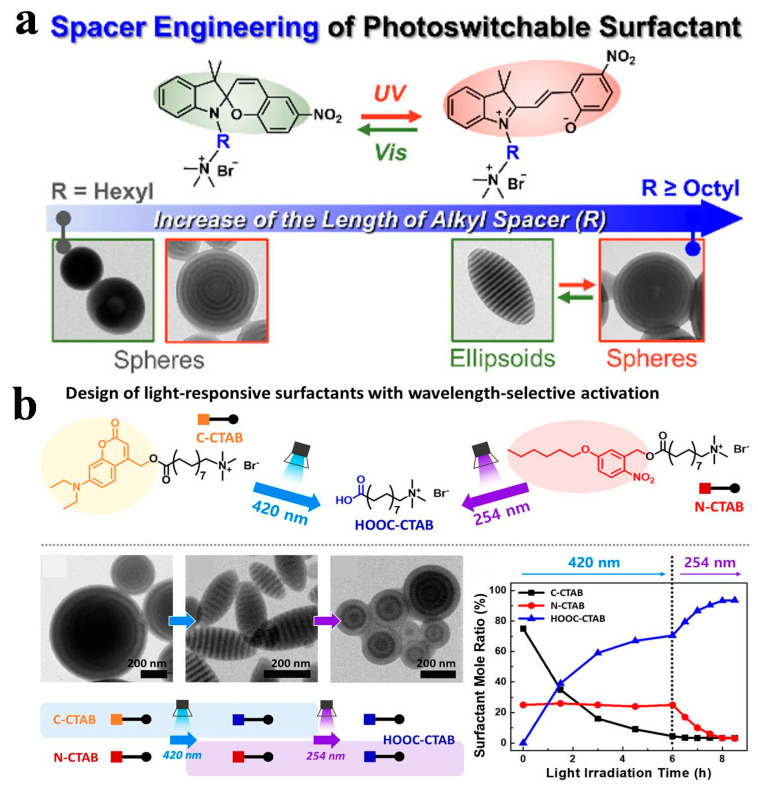
(**a**) Schematic representation of wavelength- and spacer-length-dependent morphological control in PS-*b*-P4VP mediated by SP-XTAB surfactants. Reproduced with permission [95]. Copyright 2022, ACS. (**b**) Wavelength-dependent, orthogonal triggering of morphological transitions. Reproduced with permission [96]. Copyright 2019, ACS.

In particular, the utilization of responsive surfactants provides substantial advantages in regulating the orientation and morphology of BCP [5,94]. For example, Kim et al. [95] developed a series of SP-based surfactants with varying alkyl spacer lengths, which enabled control over the photoactivity and morphology of PS-*b*-P4VP BCPs during confined self-assembly within evaporating emulsions. When the alkyl spacer length reached octane or longer, a light-driven morphological transformation occurred. Under UV light, hydrophilic open-ring merocyanine surfactants formed, while visible light induced hydrophobic closed-ring spiropyran surfactants. This transition drove the morphology to change from onion-like microspheres (with P4VP surfaces) to striped ellipsoids (with axially stacked PS and P4VP domains), as shown in Figure 6a. They also designed photoactive surfactants, namely 5-hexyloxy-2-nitrobenzyl-16-*N*,*N*,*N*-trimethylhexadecan-1-ammonium bromide (N-CTAB) and 7-(diethylamino)-4-(*N*,*N*,*N*-trimethylhexadecan-1-ammonium bromide) coumarin (C-CTAB), which undergo cleavage upon irradiation at 254 nm and 420 nm, respectively, to yield carboxylate-terminated surfactants (i.e., HOOC-CTAB). This cleavage altered their amphiphilicity and interfacial activity, thereby enabling the transformation of PS-*b*-P2VP or PS-*b*-P4VP BCP particles from spherical to prolate or oblate ellipsoidal shapes, as shown in Figure 6b [96]. In addition, Zhu et al. [97] constructed functional surfactants by introducing photoactive azobenzene groups into PS-*b*-P4VP BCPs, enabling reversible photo-responsive shape transformation of the BCP. Owing to the reversible *trans*-*cis* isomerization of the azobenzene groups under UV and visible light, the amphiphilicity and interfacial affinity of the surfactants toward different blocks were modulated. Therefore, the morphology evolved from onion-like spheres to striped ellipsoids, and finally to inverted onion-like structures.

#### 3.2.2. Nanoparticle Surfactants

**Figure 7:**
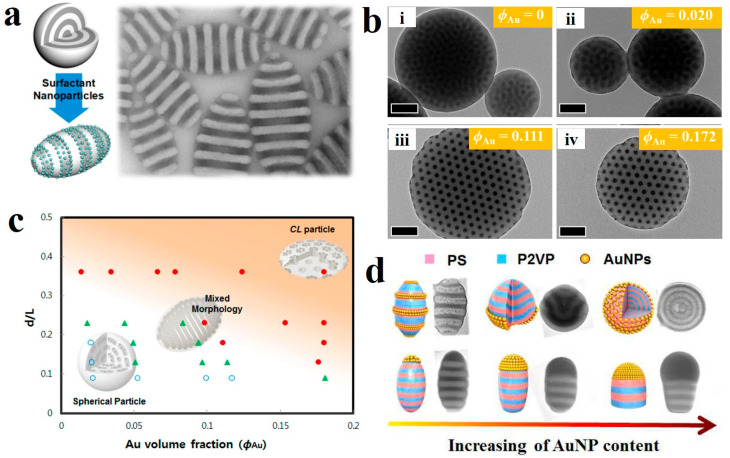
(**a**) The incorporation of Au-based surfactants induced alterations in both the shape and internal morphology of the BCP particles. Reproduced with permission [99]. Copyright 2013, ACS. (**b**) TEM characterization of Au NP-loaded PS_50k_-*b*-P4VP_13k_(PDP)_0.5_ particles at *ϕ*_Au_ = 0, 0.020, 0.111, and 0.172. The scale bars are 100 nm. (**c**) Morphological evolution of PS_50k_-*b*-P4VP_13k_(PDP)_0.5_ particles as a function of *d*/*L* versus *ϕ*_Au_, where *d* denotes Au NP diameter and *L* represents P4VP domain size. Symbols: ● (red), CL-shaped; ▲ (green), mixed morphology; ○ (blue), spherical. Reproduced with permission [98]. Copyright 2014, ACS. (**d**) Morphological evolution of AuNPs/PS-*b*-P2VP hybrid particles with increasing PS-coated AuNP content. Reproduced with permission [109]. Copyright 2017, ACS.

Surface-modified inorganic nanoparticles, such as nanospheres [98,99,100], nanorods [101,102], and graphene quantum dots [103,104,105], can serve as surfactants to effectively modulate the interactions between the polymer phase and the emulsion interface. This reduces interfacial tension, enhances the stability of the emulsion, and imparts greater resistance to deformation. For example, Hawker et al. [99] introduced Au-based surfactant nanoparticles into the PS-*b*-P2VP system, which led to a structural transformation from the traditional spherical and radial shape to a unique axial stacked lamellar ellipsoids (Figure 7a). The gold nanoparticle surfactants (Au NPs) preferentially distributed within the P2VP domains, thereby altering the oil-water interfacial interactions and resulting in neutral wetting of the PS and P2VP layers on the particle surface. Additionally, Kim et al. [98] demonstrated the application of size-controlled Au NPs in modifying PS-*b*-P4VP BCPs. The Au NPs were preferentially situated at the interface separating the surface P4VP domains from the surrounding aqueous phase. Their interfacial activity at the emulsion/water interface was crucial for the formation of CL-shaped BCP particles. By turning the size and volume fraction (*ϕ*_Au_) of the Au NPs, the internal structure and shape of the BCP particles could be effectively controlled (Figure 7b). Specifically, at *ϕ*_Au_ ≤ 0.02, most Au NPs were incorporated into the P4VP (PDP) domains, which maintained a spherical morphology. By comparison, at a higher *ϕ*_Au_ > 0.111, all particles adopted a CL shape containing porous cylindrical channels. Figure 7c summarizes the morphology of PS_50k_-*b*-P4VP_13k_ (PDP)_0.5_ particles loaded with Au nanoparticles as a function of *d*/*L* (the relative size ratio of Au NPs (*d*) over the Au NP-hosting domain (*L*), i.e., *d*/*L*) and *ϕ*_Au_. At *d*/*L* = 0.19, CL particles formed within 0.094 ≤ *ϕ*_Au_ ≤ 0.111. In contrast, at higher *d*/*L* ≈ 0.36, even minimal Au loading (*ϕ*_Au_ < 0.02) induced the CL transition, as all Au NPs segregated to the particle–water interface and acted as surfactants. Conversely, at low *d*/*L* ≈ 0.1, no transition occurred. These results clearly demonstrate that the *d*/*L* ratio governs both the internal morphology and overall shape of the BCP particles.

Compared to traditional hard nanoparticles, polymer-grafted nanoparticles combine the flexible conformation and chemical diversity of polymers with the modifiable properties of inorganic nanoparticles, resulting in unique assembly behaviors [100]. Our group [106,107,108,109] precisely controlled the morphological transformation of PS-*b*-P2VP BCPs by adjusting the concentration of Au NPs. With the increase in the incorporation of PS-modified Au NPs, the self-assembled particles transitioned morphologically from pupa-like to bud-like and finally to onion-like structures, as shown in Figure 7d. Kim et al. [110,111] also demonstrated that by regulating the molecular weight of PS in PS-grafted gold nanoparticles (Au@PS), the interactions between Au@PS and BCPs could be modulated, thereby driving morphological transformations and yielding distinct types of polymeric colloids.

**Figure 8:**
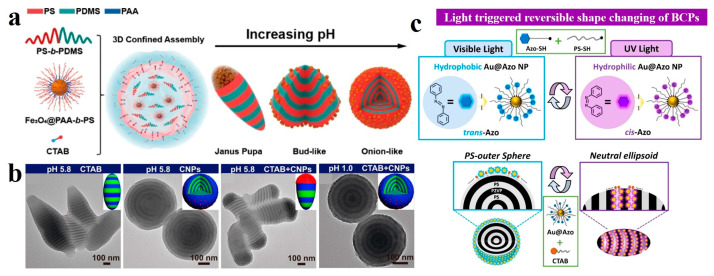
(**a**) The morphological transformation of PS-*b*-PDMS BCPs by controlling the spatial distribution of Fe_3_O_4_@PAA-*b*-PS nanoparticles. Reproduced with permission [112]. Copyright 2020, ACS. (**b**) TEM images showing pH-induced deformation of PS-*b*-PDMS microparticles: pupa-like (pH 5.8, CTAB only); onion-like (pH 5.8, CNPs only); Janus pupa-like (pH 5.8, CTAB/CNPs mixture); onion-like (pH 1.0, CTAB/CNPs mixture). Reproduced with permission [113]. Copyright 2021, ACS. (**c**) Schematic of light-responsive Au@Azo NPs and reversible morphological switching in PS-*b*-P2VP/Au@Azo hybrid particles with Au@Azo NPs/CTAB as co-surfactants. Reproduced with permission [115]. Copyright 2021, ACS.

Furthermore, incorporating responsive nanoparticles into emulsions containing BCP allows for dynamic control of interfacial interactions, facilitating the reversible morphological transformation of BCP microparticles [112,113,114]. For instance, Xu et al. [112] introduced pH-responsive poly(acrylic acid)-*block*-PS coated iron oxide nanoparticles (Fe_3_O_4_@PAA-*b*-PS) into PS-*block*-poly(dimethylsiloxane) (PS-*b*-PDMS) microparticles as a co-surfactant to regulate the interactions between oil/water. By modulating the aqueous phase pH, the positions of the Fe_3_O_4_@PAA-*b*-PS nanoparticles were altered, thereby transforming the BCP from ellipsoidal Janus pupa-like to spherical onion-like structures (Figure 8a). They further investigated the use of pH-responsive core-crosslinked polymer nanoparticles (CNPs) as co-surfactants in PS-*b*-PDMS microparticles, which imparted pH-dependent morphological changes to the system [113]. Upon decreasing the aqueous phase pH, the microparticles transitioned from Janus pupa-like to an onion-like architecture (Figure 8b). Kim et al. [115] employed azobenzene-functionalized Au nanoparticles (Au@Azo NPs) as photo-switchable surfactants to achieve wavelength-selective morphological control in PS-*b*-P2VP BCPs. The wetting layer on the surface was altered by the polarity of the Au@Azo NPs. Under visible light, the *trans*-azobenzene ligands were non-polar, resulting in onion-like microspheres with a PS outer layer of PS-*b*-P2VP. In contrast, UV irradiation induced *cis*-configuration isomerization of the azobenzene ligands, resulting in ellipsoidal particles with co-exposed PS and P2VP domains on the surface (Figure 8c).

#### 3.2.3. Polymeric Surfactants

**Figure 9:**
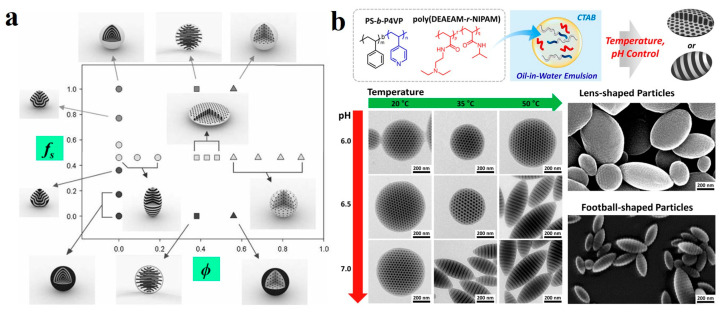
(**a**) Phase behavior of shape and internal morphology in PS-*b*-PB/hPS blend particles across the *f*_s_-*ϕ* plane. Reproduced with permission [116]. Copyright 2008, Wiley. (**b**) Chemical structures of PS-*b*-P4VP and poly(DEAEAM-*r*-NIPAM), and temperature/pH-responsive particle shape transformation between lens- and football-shaped microparticles dual-responsive surfactants. Reproduced with permission [120]. Copyright 2019, ACS.

Similar to other surfactants, amphiphilic polymers, when used as surfactants, can adsorb at the oil/water interface. By adjusting the interactions between the polymeric surfactant and the corresponding BCP blocks, they can effectively control the orientation of the BCP chains, thereby regulating the internal structure and whole shape of the BCP microparticles [93]. For instance, Yang et al. [116] synthesized PS-*block*-poly(ethylene oxide) (PS-*b*-PEO), which was selective for the PS segment, and PB-*b*-PEO, which was selective for the PB segment, as mixed polymeric surfactants for the PS-*b*-PB BCPs particles system. The volume fraction (*f*_s_) of PS blocks relative to the total volume of PS and PB blocks in the mixed surfactant system serves as a key factor governing particle shape and internal nanostructure. As *f*_s_ increases, the particle morphology undergoes a transformation from concentric multi-layered onion-like with PB exposed on outer layer, to “tulip ball” particles with alternating layers of PS and PB, to pupa-like particles with uniformly alternating PS and PB, and finally to “tulip ball” particles and concentric onion structures with PS as the outermost layer (Figure 9a). These morphological changes result from the reorganization of surfactants at the interface and alterations in the interfacial segmental affinity. By introducing homopolymer (hPS) polystyrene with a volume fraction of *ϕ*, when *f*_s_ = 0.46, flattened spherical particles with internal cylindrical structures and microspheres with PS as the continuous phase and PB spheres on the surface and inside were obtained at *ϕ* = 0.41 and *ϕ* = 0.68, respectively. Zhu et al. [117] realized reversible switching between pupa-like and onion-like morphologies by tuning the interfacial characteristics of the particles in aqueous solution. The concentration of PVA in the aqueous medium was one of the critical factors influencing the transition between these two morphologies. High concentrations of PVA favor the formation of pupa-like structures, while very low concentrations of PVA promote the preparation of onion-like particles. They [118,119] also obtained snowman-shaped Janus particles and patchy particles using PVA as the surfactant.

Researchers were also dedicated to developing BCP microparticles that can adjust their shapes and structures in response to stimuli [114,120,121]. Kim et al. [122] demonstrated temperature-driven shape and morphological transitions in polymer particles comprising PS-*b*-P4VP BCPs and a temperature-responsive poly(*N*-isopropylacrylamide) (PNIPAM) surfactant. Using a combination of PNIPAM and CTAB surfactants, ellipsoidal particles formed above the lower critical solution temperature (LCST), whereas CL-shaped particles featuring hexagonally packed cylinders emerged below the LCST. Other polymers with distinct LCST values, such as poly(*N*-*n*-propylacrylamide and poly(*N*-isopropylmethacrylamide), also enabled facile temperature-controlled morphological switching. Kim et al. [120] synthesized random copolymer surfactants based on *N*-(2-(diethylamino)ethyl)acrylamide (DEAEAM) and *N*-isopropylacrylamide (NIPAM) (poly(DEAEAM-*r*-NIPAM)). This pH/temperature dual-responsive polymeric surfactant exhibited a sharp solubility transition and underwent significant relocation within emulsified droplets in response to subtle changes in temperature and pH near physiological conditions (pH ~7.0, 25–35 °C). This behavior directly drove reversible shape transitions of PS-*b*-P4VP particles between a CL-shaped (with internal cylinders) and a striped football (with lamellae) morphology (Figure 9b). The transformation was highly reversible and could be cycled orthogonally by varying temperature and pH.

### 3.3. Additive-Mediated Control

Incorporating additives that selectively interact with one block of a copolymer offers a straightforward and effective approach to tailoring the internal structure of BCP. For example, small molecule additives [92,123,124,125,126] (8-bromooctylbenzene and bromoalkyl benzene, etc.) or polymer additives [127,128,129] (e.g., homopolymers). They favorably interact with BCP through hydrogen bonds or other strong non-covalent interactions, thereby regulating the internal structure of BCP particles. Depending on the compatibility between the additive and the polymer, during the mixing process, the additive can either enter the BCP domain or escape from the BCP, separating into an additive-rich phase.

#### 3.3.1. Small Molecule Additives

**Figure 10:**
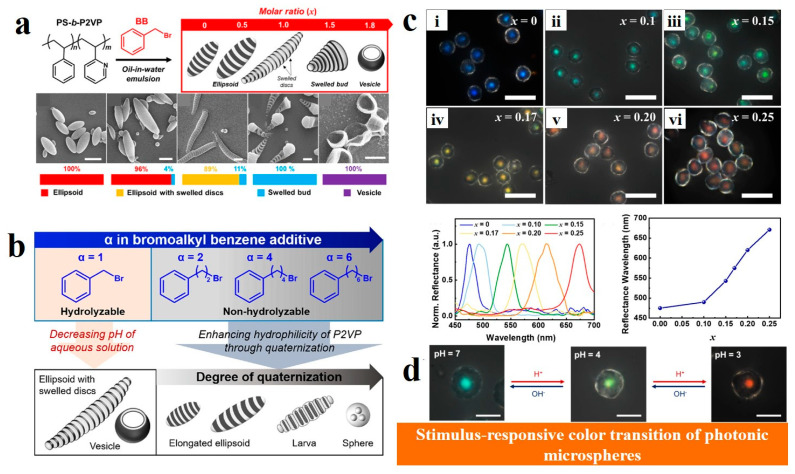
(**a**) Shape transition mechanism and SEM images of PS-*b*-P2VP(BB)*x* particles at *x* = 0 (pristine), 0.5, 1.0, 1.5, and 1.8. (**b**) Morphological evolution of BCP particles as a function of alkyl chain length. Reproduced with permission [130]. Copyright 2022, ACS. (**c**) OM images of PS-*b*-P2VP (BOB)*x* photonic microspheres at *x* = 0, 0.10, 0.15, 0.17, 0.2 and 0.25. Corresponding reflectance spectra of PS-*b*-P2VP (BOB)*x* photonic microspheres and the plot of the reflectance peak wavelength of the photonic microspheres as a function of *x*. Scale bars are 50 μm. (**d**) OM images of photonic microspheres at three different pH conditions as shown. Scale bars in OM images are 20 μm. Reproduced with permission [132]. Copyright 2025, ACS.

Organic/inorganic small molecule additives are introduced as guest molecules into BCP containing pyridyl-based monomer units, and co-assembled with BCP through non-covalent binding to achieve the regulation of the internal structure of BCP. For example, Ku et al. [130,131] investigated the morphological changes in PS-*b*-P2VP BCP particles during the quaternization reaction of bromoalkylbenzene with alkyl spacers of different lengths. Initially, benzyl bromide (BB) was used as an additive, and the molar ratio (*x*) of BB to P2VP units varied. As *x* gradually increased from 0 to 2.0, the domain spacing of PS (*D*_PS_) and P2VP (*D*_P2VP_) increased. The PS-*b*-P2VP particles underwent significant morphological evolution with increasing *x*: from lamellar axially stacked ellipsoids to ellipsoids with swollen disks (0.5 < *x* ≤ 1.0), then to swollen bud-like structures (1.0 < *x* ≤ 1.5), and finally to vesicles (1.5 < *x* ≤ 2.0), as shown in the corresponding scanning electron microscopy (SEM) images in Figure 10a. Furthermore, the alkyl spacer length of the additive was systematically varied, leading to three distinct morphological transitions: elongation of ellipsoids, larva-like particles with sandwiched lamellae, and eventual disintegration into spherical structures (Figure 10b). Kim et al. [132] synthesized a series of bromo-functionalized additives to modulate the domain spacing of PS-*b*-P2VP BCPs while maintaining the concentric lamellar onion-like morphology, thereby achieving uniform photonic microspheres with structural colors tunable across the entire visible spectrum. Among these additives, 8-bromooctylbenzene (BOB) proved most effective due to its specific quaternization reaction with P2VP chains. Simply by adjusting the molar ratio of BOB to BCP, the structural color of the photonic microspheres could be precisely controlled (Figure 10c). These photonic microspheres also exhibited reversible pH-responsive structural color changes (Figure 10d).

**Figure 11:**
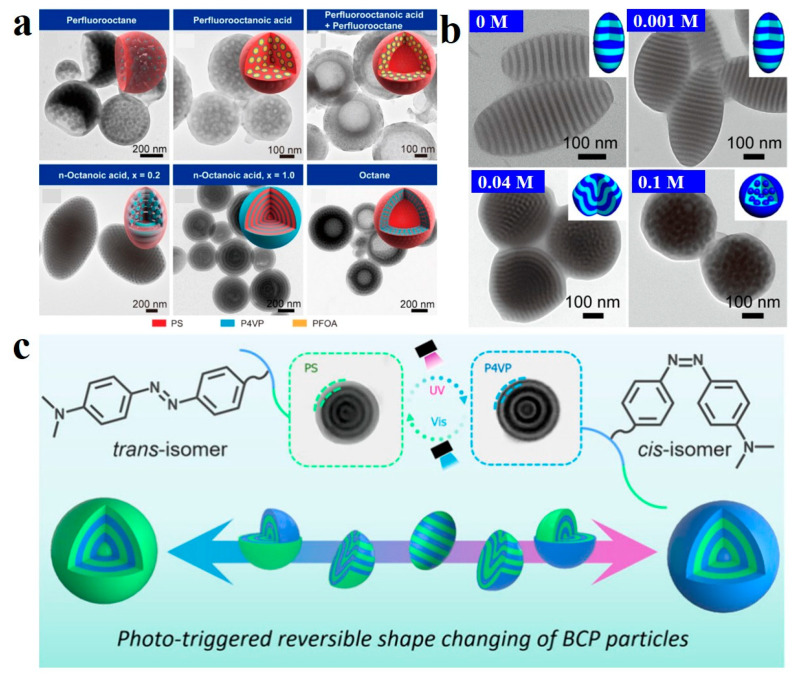
(**a**) TEM images and cartoons show the microparticle structure of blending systems having different additives. Reproduced with permission [133]. Copyright 2025, ACS. (**b**) TEM images of PS_9.8k_-*b*-P4VP_10k_-Pb(II) particles with varying Pb(II) ion concentrations. Reproduced with permission [134]. Copyright 2018, ACS. (**c**) Illustration for photodependent shape transformation with the presence of Azo-I additive. Reproduced with permission [138]. Copyright 2025, ACS.

In addition to small-molecule additives capable of undergoing quaternization reactions with block copolymers, other types, such as those enabling metal complexation or acting as non-solvent additives, can also be extensively employed in the confined co-assembly within emulsions. Xu et al. [133,134] successfully achieved microphase/macrophase separation in PS-*b*-P4VP BCPs by incorporating small-molecular fluorinated additives, utilizing competitive interactions between hydrogen bonding attraction and incompatibility-induced repulsion (Figure 11a). They also investigated metal coordination-induced three-dimensional confined self-assembly, demonstrating that the morphology of PS-*b*-P4VP could be precisely regulated by varying metal ion concentration (Figure 11b). For instance, pure PS_9.8k_-*b*-P4VP_10k_ self-assembled into elliptical pupa-like particles with alternating PS/P4VP lamellae. When the Pb (II) concentration increased to 0.04 M, the disk-like P4VP domains transformed into bent lamellar domains. At 0.1 M concentration, spherical P4VP domains formed, with this structural transformation attributed to the coordination between P4VP and metal ions.

Stimulus-responsive small molecular additives can undergo changes in overall shape and internal conformation under different conditions [135]. Additionally, they can achieve reversible structural transformation upon the removal of external stimuli, thereby offering a robust and versatile platform for the development of stimulus-responsive materials. Chang et al. [136] synthesized photoresponsive additives containing azobenzene-based bromoalkanes (i.e., Azo*x*Br) with varying lengths of alkane chains (*x* = 4, 6, 8, and 12). Through confined co-assembly of PS-*b*-P2VP and functional additives, they prepared BCP particles with light-controlled shape and structural transformation (SST) behavior. This was achieved due to the strong interaction between the nitrogen atoms in P2VP and the bromine atoms in Azo*x*Br, leading to quaternization between the additive and P2VP. By modulating the degree of quaternization, the amphiphilicity of the BCP could be finely tuned. Additionally, UV-induced *trans*-*cis* isomerization of the azobenzene groups allowed precise modulation of particle morphology and internal architecture. The BCP particles transitioned from initially striped ellipsoids to elongated ellipsoidal particles with increased structural domains, then to accordion-like particles, and eventually disassembled into small spheres, demonstrating light-driven ordered morphological transformations. They also introduced the photosensitive additive 4-hydroxyazobenzene (Azo-OH) into PS-*b*-P4VP BCPs, which realized the dual-responsive (pH and light) SST of the BCP particles [137]. The hydrogen bonding interaction between 4-hydroxyazobenzene and the 4VP structural units promoted the structural transition of the particles from an onion-like to a pupa-like morphology. Meanwhile, the UV-induced *trans*-*cis* isomerization of the azobenzene unit in Azo-OH caused the particles to evolve from a pupa-like structure to an onion-like structure with an outer P4VP layer. Since the hydrogen bonding is pH-sensitive, adjusting the pH of the solution also enabled the transition from a pupa-like to an onion-like structure. This provides promising possibilities for the development of smart materials and their applications. Very recently, Chang et al. [138] successfully achieved the co-assembly of iodine-containing azobenzene additive (Azo-I) and PS-*b*-P4VP, resulting in light-responsive BCPs with tunable morphologies. By adjusting the Azo-I content, the morphology of PS-*b*-P4VP (Azo-I) can be precisely controlled. As the Azo-I content increases, the pristine ellipsoidal particles transformed into pupa-shaped particles, and subsequently into onion-like particles with a PS outer layer. The N-I bond between the pyridine groups in the P4VP domain and Azo-I rendered the P4VP (Azo-I) domain hydrophobic, leading to the formation of onion-shaped nanoparticles with the PS domain located at the surface. However, under alternating UV and visible light irradiation, the reversible *trans*-*cis* isomerization of the Azo groups induced a hydrophobic-hydrophilic transition in the P4VP (Azo-I) domain, enabling the reversible switching of particle shape and internal nanostructure between onion-like spherical particles with a PS outer layer and inverted onion-like particles with a P4VP outer layer (Figure 11c).

#### 3.3.2. Polymer Additives

**Figure 12:**
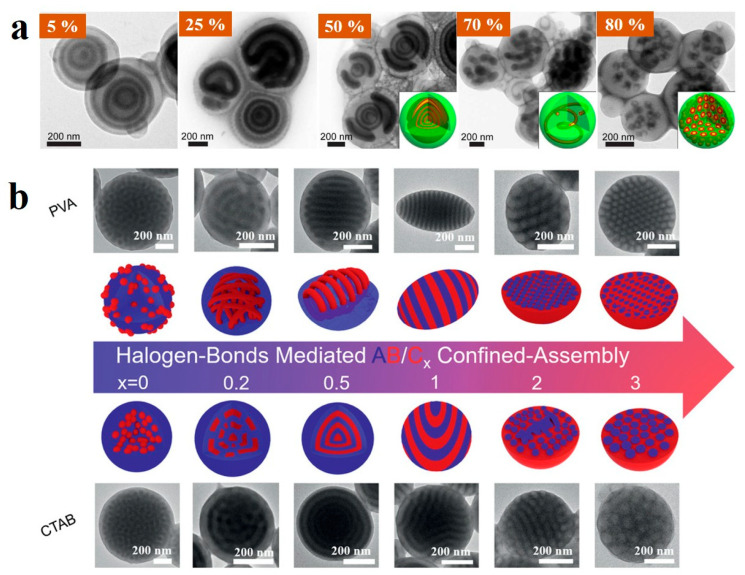
(**a**) TEM images of SIV/hPS_28K_ particles at varying hPS_28K_ weight fractions (*w*_hPS_): 5%, 25%, 50%, 70%, and 80%. Insets: schematic representations of particle structures; yellow (P2VP), red (PI), and green (PS). Reproduced with permission [142]. Copyright 2015, ACS. (**b**) Illustration of halogen bond-mediated morphological evolution in PS-*b*-P4VP/PTFIPA_34K_ blends with varying molar ratios, emulsified by PVA or CTAB. Reproduced with permission [143]. Copyright 2021, Wiley.

The introduction of additives significantly affects the Flory-Huggins interaction parameter among the blocks of BCP, thus providing an intriguing parameter for influencing the self-assembly of BCPs through homopolymer additives [139,140,141]. A clearer understanding of the impact of homopolymers can help better regulate the morphological changes in BCP particles. For example, Zhu et al. [142] achieved precise structural programming of PS-*b*-PI-*b*-P2VP (SIV) triblock copolymer particles by modulating additive-BCP interactions (Figure 12a). They demonstrated that the molecular weight of the homopolymer additive determined its miscibility with the BCP, leading to either a wet-brush or dry-brush regime. In the wet-brush regime, varying the weight fraction of hPS (*w*_hPS_) drove morphological evolution from an onion-like morphology (below 20 wt%) to onion-like particles with two domes (*w*_hPS_ = 50 wt%), and then to the formation of twisted P2VP cylinders (*w*_hPS_ = 70 wt%) and spherical P2VP domains (*w*_hPS_ = 80 wt%). In the dry-brush regime, Janus particles with distinct “head” and “cap” as well as core−shell particles were observed. Further extending this approach, they [143] introduced a halogen-bond-donating homopolymer, poly(3-(2,3,5,6-tetrafluoro-4-iodophenoxy)propyl acrylate (PTFIPA), into PS-*b*-P4VP. Halogen bonding between PTFIPA and P4VP segments increased the volume fraction of the P4VP domain, enabling an ordered morphological transition from spherical to cylindrical, to lamellar, and finally to inverse cylindrical structures by adjusting the molar ratio *x* of PTFIPA to 4VP (Figure 12b).

### 3.4. Gel Emulsion and Polymeric Hydrogels

**Figure 13:**
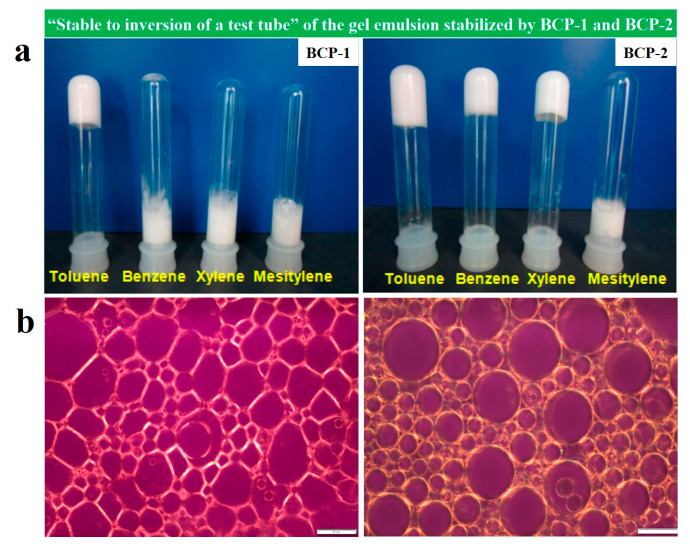
(**a**) Photographs of the gel emulsion prepared in different solvents. (**b**) Optical micrographs of gel emulsion prepared in water/toluene by using BCP-1 and BCP-2. Reproduced with permission [144]. Copyright 2018, ACS.

Polymeric hydrogels are usually 3D hydrophilic polymer networks formed by chemical or physical crosslinking. Amphiphilic BCP is used as a surfactant or stabilizer to prepare gel emulsion, which is then cross-linked by monomer polymer or BCP to obtain the polymeric hydrogel with abundant functionalities [144,145,146,147,148]. For example, Chakrabarty et al. [144] successfully prepared porous monoliths with pore sizes ranging from 5 to 50 μm using gel emulsion stabilized by the amphiphilic poly(oligo(ethylene glycol) methyl ether methacrylate)-*block*-PS (POEGMA-*b*-PS) BCP as templates. They first synthesized BCPs with different PS chain lengths (short-chain BCP-1 and long-chain BCP-2) via reversible addition-fragmentation chain transfer (RAFT) polymerization, and found that the PS chain length directly influenced the morphology, thermal behavior, and rheology of the gel emulsion. Using the “stable to inversion of a test tube” method to evaluate emulsion stability, they demonstrated that BCP-1 only formed stable gel emulsions in toluene, whereas BCP-2 formed stable emulsions in toluene, benzene, and xylene (Figure 13a). BCP-2 exhibited lower critical gelation concentrations in all solvents, indicating superior gelation capability compared to BCP-1. This enhancement was attributed to the longer PS chains providing stronger stabilization through enhanced steric repulsion in the oil phase (Figure 13b). Polymerization within the continuous oil phase (containing styrene monomer, crosslinker, and initiator) using these gel emulsions as templates yielded polystyrene monolithic adsorbents, which effectively adsorbed volatile organic compounds and exhibited excellent reusability.

**Figure 14:**
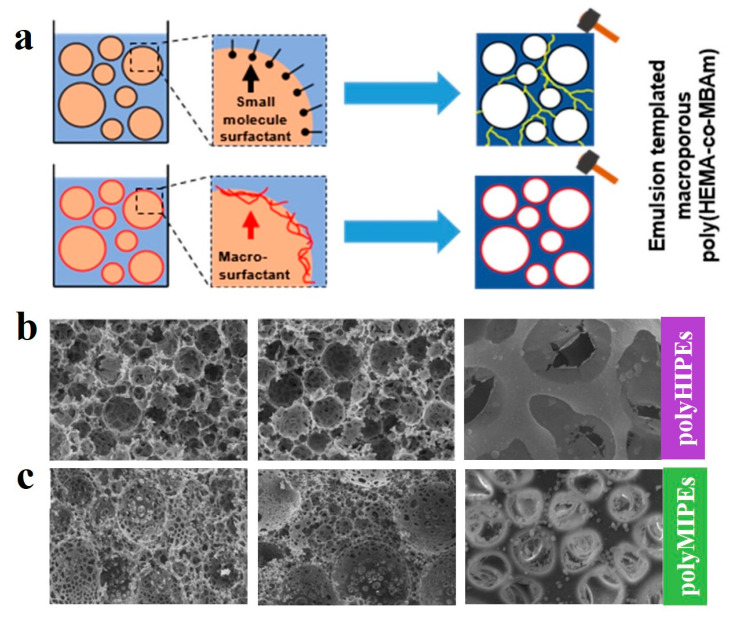
(**a**) Illustrates the functional differences between macromolecular surfactants and small-molecule surfactants. (**b**) SEM micrographs of polyHIPEs and (**c**) polyMIPEs synthesized by polymerization of emulsion templates. Reproduced with permission [145]. Copyright 2023, ACS.

Compared to small molecular additives, amphiphilic block copolymers can serve as surfactants to form high and medium internal phase emulsions (H/MIPEs), providing a robust template for the preparation of multifunctional materials. Bismarck et al. [145] reported an amphiphilic copolymer, poly(acrylamide-*co*-styrene) (P(AAm-*co*-St)), synthesized via micellar copolymerization, which functioned as a macromolecular surfactant to stabilize O/W HIPEs/MIPEs for fabricating macroporous hydrogels with excellent mechanical properties (Figure 14a). In contrast to conventional methods requiring 6–25% low molecular weight surfactants, this system stabilized toluene/water or cyclohexane/water emulsions using only 0.1 wt% copolymer with 0.01 wt% CTAB (Figure 14b,c). Using an aqueous continuous phase containing 2-hydroxyethyl methacrylate (HEMA), *N*,*N*-methylenebis (acrylamide) (MBAm) with cyclohexane as the dispersed phase, poly(HEMA-co-MBAm) porous hydrogels were prepared with 85–90% porosity and 20–50 μm pore sizes. These hydrogels exhibited outstanding mechanical properties in both dry and swollen states, over 1000% water absorption capacity reaching equilibrium within 2 h, and good resilience under cyclic compression.

For BCP-stabilized emulsion droplets, Pluronic PEO-poly(propylene oxide)-PEO (PEO-PPO-PEO, F-127) is a typical amphiphilic BCP that is commonly used and can serve as a surfactant to stabilize the emulsion droplets. For example, Silverstein et al. [146] developed a HIPE-templating strategy using reactive triblock copolymer F-127-DMA (methacrylate-end capped Pluronic PEO-poly(propylene oxide)-PEO (PEO-PPO-PEO, F-127)) to fabricate AAm-based porous hydrogels (HG-PHs) with high porosity and excellent mechanical properties. F-127-DMA served dual roles as both emulsifier and crosslinker, stabilizing O/W HIPEs while covalently anchoring the surfactant into the polymer network without requiring post-synthesis removal. The resulting HG-PHs exhibited interconnected porous structures (average pore size: 33.6–53.9 μm), low density (0.039–0.096 g/cm^3^), and microphase-separated PPO domains within pore walls. These materials demonstrated excellent compressive resistance, anti-friability, and high water absorption capacity (73–161 g/g) in dry state, with 70–80% of absorption attributed to hydrogel-swelling-driven void expansion. Incorporation of F-127-DMA also imparted temperature responsiveness, showing approximately 50% decrease in water uptake between 5 and 60 °C, with critical temperature decreasing as F-127-DMA content increased. This work provides a new approach for constructing porous hydrogels with enhanced water absorption, environmental responsiveness, and mechanical stability. Bhatia et al. [147] incorporated the amphiphilic triblock copolymer F-127 into PFOB (perfluorooctyl bromide)-based water-in-PFC (perfluorocarbon) nanoemulsion. The hydrophilic PEO segments entered the aqueous phase to stabilize the droplet cores, while the hydrophobic PPO segments anchored in the continuous PFC phase, forming bridging loops on droplet surfaces or between adjacent droplets. With increasing F-127 concentration, the size of the modified water droplets decreased while their number increased. The enhanced bridging by PPO chains strengthened the physical crosslinking density, ultimately modulating the rheological properties of the gel.

By incorporating thermos-responsive segments into the BCP surfactants used in microgel design and preparation, temperature-responsive multi-level gel materials can be achieved. For example, Kawamura et al. [148] successfully prepared core–shell microgels with a zwitterionic hydrogel core and a thermos-responsive polymer shell via inverse miniemulsion RAFT polymerization, featuring a zwitterionic poly(2-methacryloyloxyethyl phosphorylcholine) (PMPC) core and a thermos-responsive poly(oligo(ethylene glycol) methyl ether methacrylate-*co*-2-(2-methoxyethoxy)ethyl methacrylate) (P(OEGMA-*co*-MEO_2_MA)) shell. A water-soluble BCP emulsifier, P(OEGMA-*co*-MEO_2_MA)-*b*-PMPC, was first designed and synthesized to stabilize the water/chloroform interface, forming W/O emulsions. Using the emulsion droplets as nanoreactors, RAFT copolymerization of MPC and the crosslinker N,N′-methylenebisacrylamide (MBAA) yielded well-defined core–shell microgels. The resulting microgels exhibited good colloidal stability in both chloroform and water and could be readily redispersed without extensive washing. The shell polymer underwent a hydrophilic-to-hydrophobic transition around 38 °C, causing a significant decrease in dispersion transmittance and confirming its thermos-responsive behavior. Wang et al. [44] developed a one-step emulsion copolymerization method conducted at room temperature to prepare self-healable polyacrylamide-based hydrogels. Driven by hydrophobic interactions, the hydrophobic segments spontaneously aggregated and underwent phase separation during polymerization, forming hydrophobic microdomains that served as physical crosslinking points to construct a three-dimensional network. The resulting hydrogels exhibited a storage modulus up to 400 Pa and a reversible sol–gel transition at 70 °C. The incorporation of 5-acetylaminopentyl acrylate units enabled hydrogen bonding through amide groups, endowing the hydrogels with excellent self-healing capability: fractured fragments could autonomously reunite within 2 min upon contact and restore their original mechanical strength. This study provides a straightforward strategy for fabricating high-strength, physically cross-linked hydrogels with both thermos-responsive and self-healing properties.

## 4. Applications of BCP Colloids and Porous Hydrogels

BCP colloids and porous hydrogels, with their tunable morphologies and multifunctionality, demonstrate broad application potential across various fields. They are widely used in catalysis, photonic crystals, drug delivery, separation, smart materials, biomedical applications, and more.

### 4.1. Applications of BCP Colloids

#### 4.1.1. Drug Delivery

**Figure 15:**
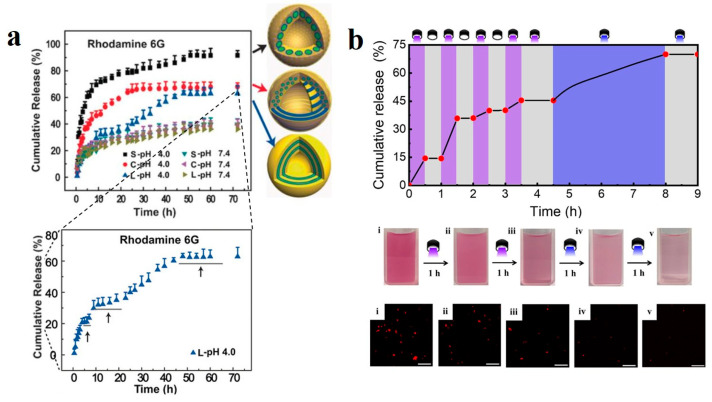
(**a**) Rhodamine 6G release profiles from capsules with varied shell structures under different pH conditions, and stepwise release from L-capsules at pH 4.0. Arrows mark the three release stages. Reproduced with permission [150]. Copyright 2016, Wiley (**b**) Cumulative NR release profiles from BCP particles under 365 nm and 420 nm irradiation, and the responding color evolution of dispersion solution and confocal laser scanning microscopy images of the BCP microparticles. The scale bars are 2 μm. Reproduced with permission [97]. Copyright 2021, ACS.

Anisotropic BCP nanocarriers, with their tailored morphologies and functionalities, enable drug delivery and controlled release, thereby enhancing therapeutic efficacy while reducing side effects. For instance, by encapsulating drug molecules within the hollow structures of BCP colloids, sustained and targeted drug delivery can be obtained. Additionally, stimulus-responsive BCP colloids (such as light-responsive or pH-responsive) enable reversible drug release illustrated a multifunctional poly(ethylene oxide)-*block*-poly(ε-caprolactone) (PEO-*b*-PCL) nanocarrier based on stimulus-responsive behavior [149]. This nanocarrier was synthesized through post-polymerization modification to produce a redox-responsive fluorescent dBCP containing the anticancer drug chlorambucil (CHL), denoted as TPE-(PEO-*b*-PCL)-S-S-CHL (P3) (TPE refers to tetraphenylethylene). The incorporation of a redox-responsive disulfide bond ensured selective drug release in the tumor microenvironment. By encapsulating methotrexate (MTX) within the BCP micelles (P3-MTX), synergistic anticancer therapy of CHL and MTX was achieved. Under high concentrations of biological reducing agent glutathione, the disulfide bond cleaves, demonstrating stimulus-responsive drug release. Tao et al. [150] reported the fabrication of PS-*b*-P4VP BCPs capsules containing high-boiling non-solvent liquid cores (e.g., hexadecane or perfluorooctane) via emulsion solvent evaporation. The resulting capsules (Figure 15a) exhibited spherical, cylindrical, or lamellar mesopores, and their structure-dependent release behavior was investigated under varying pH conditions. A faster release rate was observed as the pH decreased from 7.4 to 4.0. Recently, Chang et al. [97] introduced photoactive azobenzene groups into the confined self-assembly process of PS-*b*-P4VP BCPs and integrated Nile Red (NR) into the BCP particles to achieve photoactivated shape-switchable controlled drug release. This was because the azobenzene in the BCP particles underwent reversible *trans*-*cis* isomerization under UV irradiation, which induced a morphological transformation of the PS-*b*-P4VP microparticles. During the morphological transformation under UV and visible light irradiation, a portion of the NR was exposed to the exterior of the particles and subsequently released into the solution (Figure 15b). When the irradiation was stopped, the release ceased. This indicated that drug released from the BCP could be precisely and reversibly controlled in real time via light irradiation, occurring exclusively during its morphological transformation.

#### 4.1.2. Sensors and Catalysis

**Figure 16:**
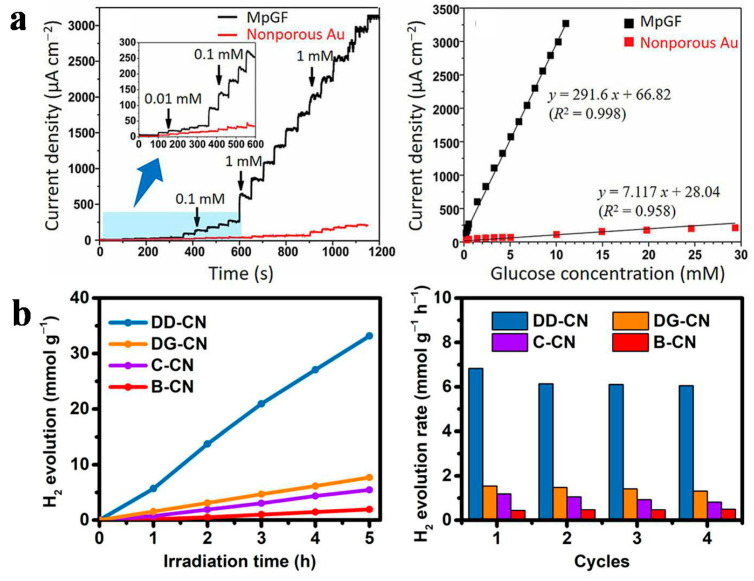
(**a**) Amperometric response (*i*–*t*) of MpGF to successive glucose additions in 0.1 M NaOH at 0.2 V (vs. Ag/AgCl), and current versus glucose concentration calibration plot. Reproduced with permission [154]. Copyright 2017, Wiley. (**b**) Photocatalytic H_2_ production rates of various CN samples (λ > 400 nm) and their cycling stability comparison. Reproduced with permission [155]. Copyright 2020, ACS.

Anisotropic BCPs can self-assemble to form ordered nanostructures such as nanowires, nanotubes, and nanofilms, which have significant applications in nanoelectronic devices, sensors, and catalysis [151,152,153]. In electrochemical sensing, BCPs with nanoporous structures facilitate the control of ion transport and enhance the interaction between the electrode and analytes, thereby improving the sensitivity and response time of signal detection. For instance, a mesoporous Au film (MpGF) electrode for glucose sensing was fabricated by electrodepositing AuCl_4_^−^ and PS-*b*-PEO onto a conductive Au/Si substrate, utilizing a micelle-assisted assembly mechanism [154]. At 40 °C, the polymer template was removed using a tetrahydrofuran solution, leading to ordered MpGF. The mesoporous structure provides more active sites, enhances the interaction between Au and adsorbed oxygen, and improves the sensitivity of glucose detection. As shown in Figure 16a, plot of the amperometric current response to the change in glucose concentration with linear regression showing sensitivity comparison between mesoporous Au and nonporous Au for glucose detection, the MpGF exhibited a 41-fold increase in sensitivity compared to non-porous Au films. Recently, Mai et al. [155] employed PS-*b*-PEO BCPs and tetraethyl orthosilicate to fabricate a double diamond (DD) structured SiO_2_ template (DD-SiO_2_) via solvent evaporation-induced self-assembly. Under vacuum conditions, melamine was sublimated and polymerized in the channels of DD-SiO_2_ to form graphitic carbon nitrides (g-CNs). The SiO_2_ template was then etched away using HF, resulting in DD-CN with an inverse DD structure. DD-CN featured two sets of 3D interconnected mesopores (average pore size of 14 nm), a specific surface area of 131 m^2^/g, and a total pore volume of 0.63 cm^3^/g. Under visible light irradiation (*λ* > 400 nm), the hydrogen production rate of DD-CN reached as high as 6831 μmol g^−1^ h^−1^, nearly twice that under full-spectrum light. This rate was approximately 16 times greater than that of non-porous bulk CN (B-CN) (420 μmol g^−1^ h^−1^), 16 times greater than that of cylindrical-pore CN (C-CN) (1188 μmol g^−1^ h^−1^), and 5 times larger than that of double-helix CN (DG-CN) (1589 μmol g^−1^ h^−1^) (Figure 16b).

#### 4.1.3. Proton Exchange Membrane Fuel Cells

Clean and renewable hydrogen fuel cells are gaining prominence, with proton exchange membrane fuel cells (PEMFCs) nearing commercialization despite persistent cost, performance, and durability limitations. Improving mass transport efficiency is crucial. BCP self-assembly in emulsion droplets enables the creation of anisotropic porous particles featuring vertically aligned channels. Carbonized versions of these particles can be directly utilized in proton exchange membrane fuel cells, opening new avenues for developing high-efficiency catalyst layers [156,157,158]. Kim et al. [157] present a method for fabricating lens-shaped mesoporous carbon (LMC) particles with vertically aligned channels (approximately 60 nm in diameter) through BCP emulsion self-assembly combined with high-temperature carbonization. By systematically adjusting the aspect ratios (ARs) of the particles (ranging from 2.1 to 6.2), their performance in PEMFCs with an ultralow platinum loading (1 wt%) was investigated. The results reveal that carbon particles with higher ARs enable denser layered stacking and improved channel alignment in the cathode, significantly enhancing the mass transport efficiency of reactants and products. Specifically, the catalyst with an AR of 6.2 achieved an initial power density of 1135 mW cm^−2^ under H_2_/O_2_ conditions and maintained a high performance of 1039 mW cm^−2^ after 30,000 cycles. This performance surpasses that of commercial Pt/C catalysts, despite using only 1/20 of the platinum loading. They also successfully fabricated ultra-low platinum-loaded (1 wt%) porous carbon microparticles with controllable channel diameters (13–63 nm) for high-performance proton exchange membrane fuel cells through BCP self-assembly combined with a two-step cross-linking and carbonization process [158]. The study demonstrated that increasing the channel diameter significantly enhances reactant mass transport efficiency. The catalyst with the largest channel diameter (63 nm) achieved an initial power density of 1230 mW cm^−2^ under H_2_/O_2_ conditions, maintaining 1120 mW cm^−2^ after 30,000 cycles. Under H_2_/air conditions, this catalyst exhibited a record-breaking performance of 51 kW g^−1^, highlighting the critical role of controlled porous carbon channel structures in improving mass transport and overall fuel cell performance.

### 4.2. Applications of Porous Hydrogels

**Figure 17:**
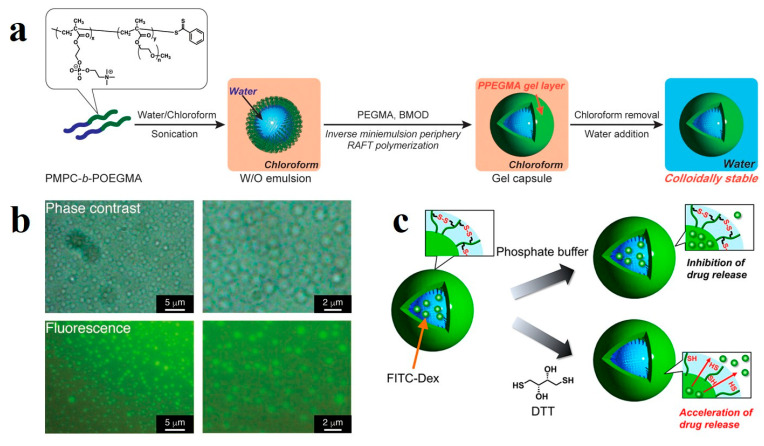
(**a**) Schematic of gel capsule preparation via IMEPP using a PMPC-*b*-POEGMA-stabilized W/O emulsion template. (**b**) Phase contrast and fluorescence images of W/O emulsions after sonication for 1 h. (**c**) Enhanced reductive-responsive release of FITC-Dex from PPEGMA capsules. Reproduced with permission [159]. Copyright 2019, ACS.

Porous hydrogels are a fascinating class of materials that combine the absorbent, soft, and biocompatible properties of hydrogels with a highly interconnected network of pores. This porosity drastically enhances their functionality, making them indispensable in a wide range of advanced applications. For example, Kawamura and et al. [159] reported the preparation method and drug release behavior of reductively responsive gel capsules based on a water-soluble zwitterionic BCP, PMPC-*b*-POEGMA. The BCP was synthesized via RAFT polymerization, in which the PMPC block is strongly hydrophilic and the POEGMA block is amphiphilic, enabling it to act as an emulsifier stabilizing W/O emulsions in a water-chloroform biphasic system (Figure 17a,b). Based on this, inverse miniemulsion periphery RAFT polymerization (IMEPP) was employed to copolymerize PEGMA with a disulfide-containing crosslinker, bis(2-methacryloyl)oxyethyl disulfide (BMOD), at the surface of the emulsion droplets, resulting in the formation of reductively responsive PPEGMA gel capsules. These capsules can be stably dispersed in both water and chloroform without additional hydrophilic modification. Using fluorescein-conjugated dextran (FITC-Dex) as a model drug, its release behavior in a buffer containing the reducing agent dithiothreitol (DTT) (Figure 17c) was investigated. The results showed a significantly accelerated drug release rate under reducing conditions, attributed to the cleavage of disulfide bonds leading to the relaxation of the gel network structure, offering a promising platform for smart drug carriers in biomedical applications such as protein and gene delivery.

**Figure 18:**
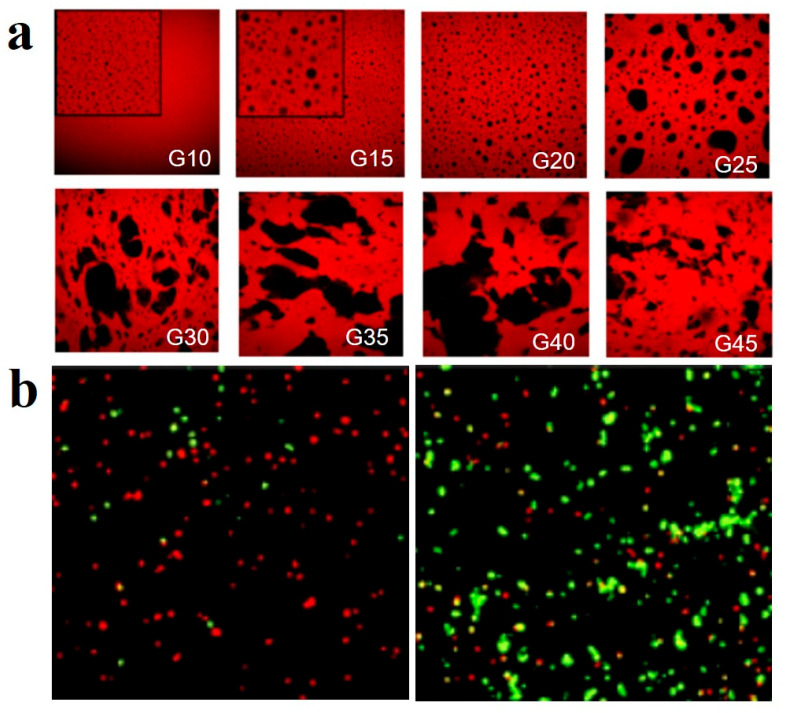
(**a**) CLSM images of biphasic mixtures with different compositions. (**b**) CLSM images of cryopreserved GStemHep encapsulation in tPEO after 3 h and in the G30 hydrogel after 24 h. Reproduced with permission [41]. Copyright 2023, ACS.

Nicol et al. [41] developed a biphasic photocrosslinkable hydrogel based on PEO and gelatin (GEL), with tunable morphology suitable for hepatic progenitor cell (GStemHep) encapsulation. The hydrogel was formed by mixing aqueous solutions of the two polymers to create a water-in-water emulsion, which was then stabilized via photopolymerization. In this system, the PEO-based triblock copolymer (tPEO) constituted the continuous phase, providing mechanical support, while GEL formed the dispersed phase, creating macroporous structures. By adjusting the volume fraction of GEL (5–45%), various morphologies, from isolated spherical pores to interpenetrating interconnected pores, could be achieved, as shown in the confocal laser scanning microscopy (CLSM) in Figure 18a. When the gelatin volume fraction approached 50%, the addition of a small amount of xanthan effectively stabilized the co-continuous structure, resulting in self-standing hydrogels. The hydrogel exhibits a Young’s modulus between 5 and 30 kPa, comparable to that of soft tissues, and shows no swelling or degradation under physiological conditions. Preliminary cell experiments indicated low GStemHep viability in monophasic tPEO hydrogels, whereas significantly improved cell survival was observed in tPEO/GEL macroporous hydrogels (Figure 18b). In vivo experiments in immunocompetent mice showed that implanted hydrogels encapsulating GStemHep sustainably secreted human alpha-fetoprotein (AFP), supporting their potential for cell therapy and tissue engineering.

## 5. Conclusions and Perspective

Emulsion droplets that have flexible and deformable interfaces can offer a versatile confined assembly environment for the fabrication of novel functional BCP colloidal microparticles. Additionally, concentrated colloidal emulsions stabilized by BCPs can serve as gel emulsions and internal porous templates for the preparation of porous materials, such as hydrogels and other functional porous materials. Therefore, the preparation methods of emulsions and their applications in functional materials hold significant research implications and practical value. This review begins by introducing current methods for preparing monodisperse emulsions, including microfluidic and SPG membrane emulsification techniques, which enable the production of uniform emulsion droplets with controllable sizes, thereby providing high-quality templates for the fabrication of functional materials. When BCPs are confined within emulsion droplets, the specific internal nanostructures of resulting BCP colloidal microparticles can be effectively tailored by adjusting polymer intrinsic properties, surfactant types, and additives. Furthermore, when amphiphilic BCPs are employed as surfactants to stabilize emulsion droplets, gel emulsion can be obtained, which can be utilized as a highly effective internal emulsion template for the production of hydrogels and functional porous polymer materials. Finally, we conclude by highlighting the potential applications of these engineered colloidal microparticles and hydrogel porous materials in diverse areas, including drug release, systems, fuel cells, sensors and catalysis.

Despite significant advancements in the preparation and application of BCP colloids and hydrogels stabilized by amphiphilic BCPs, several challenges remain. For instance, achieving large-scale production of emulsion droplets or gel emulsions is still a hurdle. While membrane emulsification techniques can produce monodisperse particles, the yield of specific shapes (such as tetrahedra) is extremely low, and photolithography templates are costly. Additionally, there is a need to further expand the applications of BCP colloids, gel emulsion templates and porous hydrogels across various fields. Currently, most research focuses on the preparation of functional polymeric materials responsive to temperature, pH, or light. Further development of other functional stimulus-responsive colloids and hydrogels could extend their potential applications in areas such as biomedicine and energy.

## Data Availability

No new data were created or analyzed in this study.
